# The Use of Non-Variant Sites to Improve the Clinical Assessment of Whole-Genome Sequence Data

**DOI:** 10.1371/journal.pone.0132180

**Published:** 2015-07-06

**Authors:** Alberto Ferrarini, Luciano Xumerle, Francesca Griggio, Marianna Garonzi, Chiara Cantaloni, Cesare Centomo, Sergio Marin Vargas, Patrick Descombes, Julien Marquis, Sebastiano Collino, Claudio Franceschi, Paolo Garagnani, Benjamin A. Salisbury, John Max Harvey, Massimo Delledonne

**Affiliations:** 1 Functional Genomics Center, Department of Biotechnology, University of Verona, 37134, Verona, Italy; 2 Personal Genomics s.r.l, Strada le Grazie 15, 37134, Verona, Italy; 3 Functional Genomics, Nestlé Institute of Health Sciences SA, EPFL Innovation Park, bâtiment G, 1015, Lausanne, Switzerland; 4 Molecular Biomarkers, Nestlé Institute of Health Sciences SA, EPFL Innovation Park, bâtiment H, 1015, Lausanne, Switzerland; 5 Department of Experimental, Diagnostic and Specialty Medicine Experimental Pathology, University of Bologna, Via S. Giacomo 12, 40126, Bologna, Italy; 6 Interdepartmental Centre “L. Galvani” (CIG), University of Bologna, Piazza di Porta S. Donato 1, 40126, Bologna, Italy; 7 IRCCS, Institute of Neurological Sciences of Bologna, Ospedale Bellaria, Via Altura 3, 40139, Bologna, Italy; 8 Center for Applied Biomedical Research, St. Orsola-Malpighi University Hospital, 40138, Bologna, Italy; 9 Knome Inc., Waltham, Massachusetts, 02451, United States of America; Oslo University Hospital, NORWAY

## Abstract

Genetic testing, which is now a routine part of clinical practice and disease management protocols, is often based on the assessment of small panels of variants or genes. On the other hand, continuous improvements in the speed and per-base costs of sequencing have now made whole exome sequencing (WES) and whole genome sequencing (WGS) viable strategies for targeted or complete genetic analysis, respectively. Standard WGS/WES data analytical workflows generally rely on calling of sequence variants respect to the reference genome sequence. However, the reference genome sequence contains a large number of sites represented by rare alleles, by known pathogenic alleles and by alleles strongly associated to disease by GWAS. It’s thus critical, for clinical applications of WGS and WES, to interpret whether non-variant sites are homozygous for the reference allele or if the corresponding genotype cannot be reliably called. Here we show that an alternative analytical approach based on the analysis of both variant and non-variant sites from WGS data allows to genotype more than 92% of sites corresponding to known SNPs compared to 6% genotyped by standard variant analysis. These include homozygous reference sites of clinical interest, thus leading to a broad and comprehensive characterization of variation necessary to an accurate evaluation of disease risk. Altogether, our findings indicate that characterization of both variant and non-variant clinically informative sites in the genome is necessary to allow an accurate clinical assessment of a personal genome. Finally, we propose a highly efficient extended VCF (eVCF) file format which allows to store genotype calls for sites of clinical interest while remaining compatible with current variant interpretation software.

## Introduction

More than 24,000 tests for 5000 conditions are currently available on the NCBI Genetic Testing Registry (GTR) [http://www.ncbi.nlm.nih.gov/gtr/] and genetic testing has grown from a niche specialty for rare disorders to routine clinical practice [1,2]. These include groundbreaking examples of newborn screening tests for highly-penetrant disorders, diagnostic and carrier testing for inherited disorders, risk stratification/screening, and pharmacogenetic testing to guide individual drug selection and dosage regimens. For some diseases, genetic tests have been woven into clinical practice and disease management. For example, germline *BRCA1*/*BRCA2* genotyping is used to determine susceptibility to breast and ovarian cancer, and human leukocyte antigen (HLA) genotyping is used to determine susceptibility to celiac disease. Genetic tests are also used for therapeutic management, e.g. mutation testing in the epidermal growth factor receptor gene (*EGFR*) is used to predict the efficacy of the EGFR-targeting antibodies gefitinib and erlotinib, which are indicated for non-small cell lung cancer [3,4].

Genetic tests are generally based on the analysis of individual or small panels of variants and are carried out using classical techniques such as allele-specific PCR or ligation-based SNP genotyping assays as implemented, for example, in the Illumina VeraCode platform, Luminex, Sequenom and TaqMan assays. However, these tests are limited to well-characterized mutations and are often followed by sequencing of the complete gene(s). For example, approximately 1500 different mutations have been identified in the cystic fibrosis transmembrane conductance regulator gene (*CFTR*). Potential cystic fibrosis patients are initially screened using a standard genetic test based on a panel of the 23 most common mutations [5,6] and if these tests are negative then the complete *CFTR* gene is sequenced to identify rare or uncharacterized causal mutations. Bidirectional Sanger sequencing is still considered the ‘gold standard’ in clinical genetic testing [7,8] but several amplicons must be sequenced in order to cover the target region, and this approach has a low sensitivity which becomes limiting when the sample tissue is scarce, e.g. in the analysis of tumors where the percentage of cells carrying the causal mutation must be at least 15–20% for efficient detection.

With the introduction of next-generation sequencing (NGS) technologies, the length of DNA to be sequenced is no longer a limit, and human genome diagnostics now embraces the targeted sequencing of individual genes and multigene panels [9]. This has given geneticists the ability to increase the clinical sensitivity for many tests and to investigate the substantial contribution of unique and rare variants to disease. A number of NGS-based panels for clinical cancer genomic profiling have been developed [10] and commercial kits are currently used in clinical practice and as clinical trial assays (CTAs) to guide patients towards the most appropriate treatment [11–13]. Clinical multigene NGS panels comprising from approximately 10 up to several hundred genes are also available for immunological, neurological, sensory, cardiac, neuromuscular and metabolic disorders.

The declining cost and improving coverage of whole exome sequencing (WES) and whole genome sequencing (WGS) means that these techniques are now useful alternatives to gene panels, and are becoming increasingly prevalent in the clinic. Panels of molecular assays can be replaced with “virtual panels” of genes or variants that are used to query the WES/WGS data. A clear advantage of the WES/WGS approach is that it can be used to test as many virtual panels for different diseases and responses as necessary without the need to conduct independent assays for each genetic test. Furthermore, as well as identifying the variants underlying the target disease, WES/WGS can also identify secondary or incidental variants that may be clinically or personally relevant [14]. Finally, WES/WGS is future-oriented because as soon as new disease markers are identified these can be added to the “virtual panel” and extant sequence data can be reanalyzed accordingly.

WES requires highly-redundant coverage because its uniformity is affected by GC content, due to the impact of this property on the efficiency of both amplification and oligonucleotide hybridization [15]. In contrast, WGS achieves more uniform and comprehensive coverage even at a read depth of only 30x, including regions generally missed by targeted sequencing [16], based on the increasing efficiency of PCR-free library preparation methods [17]. WES and WGS data are typically stored as variant call format (VCF) files, which have been widely adopted by the community [18]. VCF was developed to support large-scale genotyping and DNA sequencing projects, specifically the 1000 Genomes Project, because other genetic data storage formats such as general feature format (GFF) contain complete genetic data, much of which is redundant because it is shared across genomes, whereas VCF stores only the variants relative to a reference genome sequence. However, this format was developed primarily to represent human genetic variation and not to make causal links between variants and human disease.

We evaluated the genetic predisposition to heart disease in a patient with abnormal cardiac electric activity, by comparing base calling from WGS data with the results obtained from a commercial genetic testing kit (MI Risk plus) based on Illumina VeraCode technology. This assessment confirmed the power of WGS analysis but revealed that standard variant calling pipelines based on VCF are limited in their ability to call the patient genotype because they cannot call the genotype in loci homozygous or hemizygous for the reference allele. We addressed these limitations by applying an alternative approach based on the use of the genome VCF (gVCF) file and showed that it allows a much broader characterization of known variants for individuals undergoing clinical care.

## Materials and Methods

### Ethics statement

The study has been approved by the "Comitato Etico per la Sperimentazione Clinica (CESC) delle province di Verona e Rovigo" ethic board and all clinical investigation have been conducted according to the principles expressed in the Declaration of Helsinki. Following a detailed explanation of the study, written informed consent was obtained from a patient with a cardiac QT interval (QTc) of 504 ms.

### Genotyping using the MI Risk Plus kit and SNP microarray

Genomic DNA was isolated from buccal swabs using the QIAamp DNA mini kit (Qiagen) according to the manufacturer’s instructions and was quantified using a Qubit fluorimetric assay (Invitrogen). The sample was processed using the MI Risk Plus kit (Personal Genomics S.r.l.) based on the VeraCode GoldenGate Genotyping Assay kit (Illumina Inc.) and a custom Oligo Assay Pool (Illumina Inc.) based on 96 SNPs related to coronary artery disease, according to the manufacturer’s instructions. Genomic DNA (2.5 μg) was sent to HelmholtzZentrum München for genotyping using an Illumina Omni 2.5 microarray followed by analysis with GenomeStudio (Illumina Inc.) using the original Illumina cluster and manifest files (HumanOmni2.5-4v1_H.egt and HumanOmni2.5-4v1_H.bpm). Genotypes were exported using the Report Wizard in GenomeStudio Standard format. The output was then converted to VCF format and inconsistencies between the expected alleles based on dbSNP (build 141) and alleles declared in the Illumina manifest files were corrected using an in-house script.

### Library preparation and whole-genome sequencing

Control sample NA12892 was obtained from the NIGMS Human Genetic Cell Repository at the Coriell Institute for Medical Research. Patient genomic DNA was isolated from 10 ml of whole blood using standard salting-out method. Genomic DNA libraries were prepared using the TruSeq DNA Sample Preparation Kit (Illumina) and TruSeq DNA PCR-Free Sample Preparation Kit (Illumina) according to the manufacturer’s instructions. The libraries were sequenced on a HiSeq sequencing system (Illumina) generating 100 bp paired-end reads.

### Reads alignment and variant calling

FASTQ files obtained from the sequencing of NA12892 libraries obtained with the standard library preparation protocol were downloaded from the 1,000 Genomes Project ftp repository at EBI (ftp://ftp.1000genomes.ebi.ac.uk). Reads generated by genome sequencing or downloaded from 1000 Genomes Project repository were aligned to the human hg19 reference genome sequence using Isaac Genome Alignment Software, and variant calling was performed using Isaac Variant Caller with default parameters [19].

### Variants annotation and data integration

The GWAScat database of snp-trait associations was downloaded (2014-08-19) from the National Human Genome Research Institute (NHGRI) website (http://www.genome.gov/gwastudies/) and coordinates were mapped from hg38 to hg19 using dbSNP rs code IDs. Genotyping data from MI Risk plus, the Human Omni 2.5 microarray and genome sequencing together with dbSNP (build 141), ClinVar (version 4.9.2014) and GWAScat (2014-08-19) were imported into a relational database (PostgreSQL) for fast data querying and retrieval.

### Format conversion

The gVCF format was converted to eVCF by taking vcf files representing known variant sites from dbSNP (build 141) and converting them with the in-house script gvcf2evcf (http://ddlab.sci.univr.it/files/gvcf2evcf). Each eVCF was imported without modification into the knoSys 3.1 software-hardware analysis platform (Knome Inc.), into SNP & Variation Suite (SVS) 8.2.1 (Golden Helix Inc.) and into Variant Effect Predictor (VEP release 77). Each eVCF file was converted to ANNOVAR standard input format with an in-house script (http://ddlab.sci.univr.it/files/evcf2annovar).

## Results

### Comparison of standard and PCR-free library preparation protocols for WGS

**Fig 1:**
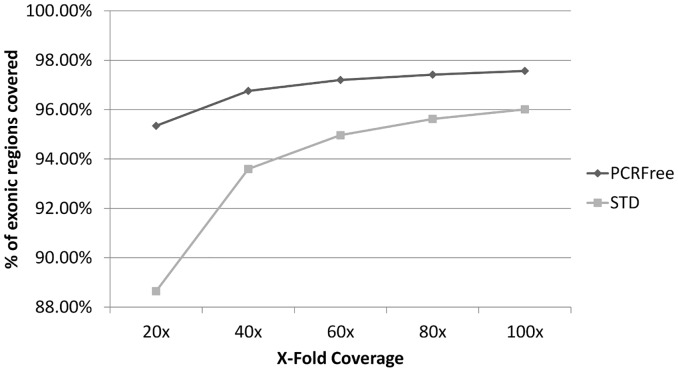
Exonic regions coverage. Percentage of exonic regions covered at a read depth ≥ 5, an alignment score ≥ 10, a basecall quality ≥ 10 from WGS subsets of the original full set with different average X-fold coverage values.

We compared WGS sequence data from the NA12892 CEU individual of European ancestry produced using a standard library preparation protocol (STD) and the PCR-free library preparation protocol (PCRFree). The two filtered datasets were normalized to 106 estimated X-fold coverage to allow unbiased comparison. In both cases, more than 90% of the reads were mapped to the hg19 reference sequence (STD 93.0%, PCRFree 98.5%) and the two datasets showed overall good uniformity of coverage (S1 Fig). The PCRFree protocol achieved a reduction of the number of gaps from 134,001 to 78,898, defined as regions longer than 10 bp with a low read depth (< 5), low alignment score (Q score < 10) and low basecall quality (Q < 10). This accounted for a reduction in the number of bases included in gaps from 102.7 Mbp to 54.7 Mbp. The PCRFree dataset also achieved a significant increase in the average read depth and coverage uniformity in regions with high (≥75%) and extreme (≥85%) GC content, and in the presence of repeated AT dinucleotides, ranging from 151% to 664 (S2 and S3 Figs). The typical WGS coverage generated by the Illumina HiSeq X-Ten run is 30–40 X-fold, so we evaluated the percentage of exonic regions covered (read depth > 5, mapping score > 10 and basecall quality score > 10) by datasets with different average coverage values ranging from 20 to 100 X-fold generated by sub-sampling the full available dataset. A previous study, based on sequencing of illumina standard libraries, reported that an average mapped depth of 50 X-fold is required to produce confident genotype calls for >80% of the exome and showed that a 40–45 X-fold coverage to detect most of SNPs [20]. We found that the 40 X-fold PCRFree dataset covered more than 96.7% of the exonic regions with the given thresholds and that increasing the average genome coverage up to 100 X-fold only increased the percentage of exonic regions covered by 0.81% (Fig 1). Based on these results, we decided to use the PCRFree protocol for library preparation and perform WGS at a target mean coverage of 35–40 X-fold. It is worth noting that an higher mean X-fold coverage might be required to genotype INDELs reliably. Indeed, a recent work by Fang *et al*. showed that 60 X-fold mean coverage is needed to recover 95% of INDELs [21].

### Concordance of WGS with MI Risk plus

A patient showing a disturbance in cardiac electrical homeostasis associated with an increased risk of sudden cardiac death (QTc = 504) was genotyped with MI Risk plus, an Illumina VeraCode-based assay of 96 SNPs associated with cardiac diseases. The MI Risk plus kit successfully genotyped 95 SNPs using material from the patient (S1 Table). PCRFree WGS was then used to call variants in the patient’s VCF file generated from 37X WGS (557,165,871 fragments, 100 bp x 2) and the two datasets were compared. Only 54 of the 96 SNPs were represented in the VCF file, two of which were genotyped as homozygous references by the kit but were called as heterozygous variants in the VCF file (Table 1 and S1 Table). Sanger sequencing confirmed as correct the two genotypes detected by WGS.

**Table 1:** Concordance of genotypes represented in VCF and gVCF files with those detected by the MI RISK Plus kit.

	VCF	gVCF
**SNPs genotyped**	55	96
***Concordant with MI RISK Plus***	52	92
***Non-concordant with MI RISK Plus***	2	2
***Not called by MI RISK Plus***	1	1

The 41 SNPs that were not present in the VCF file were detected as homozygous reference genotypes by the MI Risk plus kit. Sites that are not detected by variant analysis are generally inferred as homozygous reference sites, but in clinical diagnostics it is mandatory to distinguish clear homozygous reference sites from those not called as variant because the quality of the call is below the filter threshold. We therefore generated a gVCF file from the sequence alignments representing all the sites on the genome with associated calling confidence score metrics (https://sites.google.com/site/gvcftools/). Each of the 41 SNPs that were not represented in the VCF file were found in the high-confidence invariant block records of the gVCF file, representing homozygous reference regions (Table 1).

### Concordance with SNP genotyping arrays and GWAS data

Many of the disease-relevant variants identified during the last 10 years have been found by SNP genotyping in genome-wide association (GWAS) experiments [22,23]. We therefore compared the patient genotype data obtained by WGS analysis with that obtained by screening the Illumina HumanOmni2.5 microarray. Microarray analysis revealed genotypes for 2,356,959 SNPs among 2,366,934 targets whereas only 671,861 (28.6%) positions were called in the VCF file, 3279 of which were not genotyped by the microarray (S4 Fig). Analysis of high-confidence invariant block records in the gVCF file allowed us to assign a homozygous reference genotype to an additional 1,641,412 SNPs targeted by the microarray, thus increasing the total number of targeted SNPs detected by WGS to 2,313,273, among which 9332 were not genotyped by the array. Thus, gVCF-based genotype calling from the 37X WGS achieved high sensitivity (97.81%) and high specificity (99.96%) compared with the microarray, and the concordance of genotypes was 99.3%. We identified 14,948 variants on the microarray that were called as invariant sites from WGS data, and 538 variants based on WGS data that were invariant on the microarray. After removing microarray data from 84,774 probes with INDELs or SNPs 1–10 bp away from the targeted SNPs (S5 Fig), the number of variant sites on the microarray that were called as invariant from WGS data was reduced to 2602 and the sensitivity increased to 99.6%.

Many common variants have been assessed in GWAS for their association with human traits such as increased risk of either protection from disease or response to drugs [23,24]. The NHGRI GWAS Catalog (downloaded on 2014-08-19) contains 13,562 associations between variants and human traits, including 2070 protective alleles and 1142 risk alleles that are potentially clinically relevant and present in the hg19 reference genome (Table 2) and therefore are not reported in a VCF file. Indeed, analysis of our patient’s VCF file genotyped only 6700 sites (Table 2), whereas the gVCF file successfully genotyped 13,357 sites (98.4%) including 6657 homozygous reference sites. For the remaining 205 sites in GWAScat, it was not possible to infer a genotype with confidence. We then focused on 3212 sites with an associated odds ratio (OR) and an annotated risk allele. Among the 2070 sites where the reference contains the protective allele (median OR = 0.54), 2040 could be genotyped from the gVCF file (S2 Table), among which 1036 were homozygous reference sites and therefore were not called in the VCF file. Among the 1142 sites where the reference contains the risk allele (median OR = 1.86), 1125 were genotyped in the gVCF file (S3 Table), including 429 homozygous reference sites not reported in the VCF file.

**Table 2:** Genotyping of GWAS catalog sites using the VCF and gVCF file formats and the number of homozygous reference sites and no-calls based on WGS data.

	Total sites	VCF	gVCF	Homozygous reference	No-calls
**GWAScat**	13,562	6,700	13,357	6657	205
**GWAScat protective allele on ref**	2070	1004	2040	1036	30
**GWAScat risk allele on ref**	1142	696	1125	429	17

### WGS analysis of variant and invariant sites with known clinical associations

Although concordance with common variant arrays helps to evaluate the ability of WGS to detect variant and invariant genotypes, the comparison is limited to a small proportion of the 53,615,998 known SNPs represented in dbSNP build 141, many of which are associated with human phenotypes as reported in ClinVar [25]. To perform a reliable and comprehensive clinical evaluation of a genome it’s important to genotype not only variant sites but also homozygous reference sites of potential clinical interest. Indeed, we found that a large number of sites is represented by a minor allele in the hg19 reference genome (S4 Table), out of which 88,428 are rare (MAF < 1%) in the general population and 2765 were detected as homozygous for the reference allele in our patient (Table 3 and S5 Table). Rare variants are potentially dangerous candidates which may, for example, impair gene functionality. It is worth noting that among variants represented by a minor allele in the reference 285 low-frequency alleles with a MAF < 5% and 132 rare alleles with a MAF < 1% were located in exons.

**Table 3:** Genotyping of known SNPs from dbSNP 141 using the VCF and gVCF file formats and the number of homozygous reference sites and no-calls based on WGS data.

	Total sites	VCF	gVCF	Homozygous reference (in exons)	No-calls (in exons)
**Known SNPs (dbSNP 141)**	53,615,998	3,301,013	49,651,226	46,350,213 (2,241,575)	3,964,772 (114,639)
**Known v. with minor allele on reference**	1,836,144	1,467,990	1,681,958	213,968 (3573)	154,186 (2154)
**Known v. with minor allele on reference (MAF <5%)**	242,213	219,675	225,333	5664 (80)	16,880 (285)
**Known v. with minor allele on reference (MAF <1%)**	88,428	80,386	83,148	2765 (49)	5280 (132)

Of the 19,314 sites for which ClinVar (version 4.9.2014) has an allele annotated as pathogenic or as drug response, 96% could be read from the gVCF in contrast to only 9% from the standard VCF (Table 4 and S6 Table). The patient carried a homozygous reference genotype for 11 pathogenic and 1 drug-response sites represented in the hg19 reference genome by a risk allele, which could therefore be detected only by the analysis of the gVCF file (Table 4 and S6 Table).

**Table 4:** Genotyping of known SNPs from ClinVar using the VCF and gVCF file formats and the number of homozygous reference sites and no-calls based on WGS data.

	Total sites	VCF	gVCF	Homozygous reference	No-calls
**ClinVar**	87,087	1,810	83,675	81,865	3412
**ClinVar pathogenic**	19,280	62	18,965	18,903	315
**ClinVar drug response**	34	7	34	27	0
**ClinVar pathogenic, risk allele on ref.**	29	15	26	11	3
**ClinVar drug response, risk allele on ref.**	3	2	3	1	0

### Construction of an extended VCF file

The gVCF file format is not supported by most current variant analysis platforms, including Annovar, Variant Effect Predictor (VEP) and SnpEff [26–28] (https://atgu.mgh.harvard.edu/plinkseq/) and the file size often exceeds 1.5 Gb for a WGS dataset and 150 Mb for a WES dataset. To produce a file format compatible with current analysis pipelines but containing information relevant for clinical applications, we built a VCF file with extended content, and named it extended VCF (eVCF). The eVCF file contains the genotypes at all the variant positions identified by variant analysis plus the genotype at all positions defined in a set of interest, for example dbSNP. The version of dbSNP (or of any other list of variants) used to generate the eVCF file is indicated in the file header. We also included annotations “known”, “gwascat_id” and “odds_ratio” in the INFO field to allow the filtering for disease-associated variants. Typical sizes and contents for BAM, standard VCF, gVCF and eVCF files are compared in Table 5. We tested the compatibility of the eVCF format with two commercial platforms for variant annotation and interpretation, knoSys 3.1 (Knome Inc.) and SVS 8.2.1 (Golden Helix Inc.), and with two open source and widely used platforms, ANNOVAR (version 2014Nov12) [26] and VEP release 77 [27]. All four packages were compatible with the eVCF file and imported most of the genotypes (Table 6). Finally we compared the number of known SNPs (dbSNP build 141), ClinVar-annotated SNPs and GWAScat SNPs genotyped by the eVCF compared to standard VCF and gVCF. In all cases, the eVCF genotyped the same number of sites as the gVCF file (Table 7).

**Table 5:** Comparison of the content and size of different standard file formats for the storage of genomic data.

File format	Content	Size
**BAM (37 X-fold coverage)**	Read alignments	81 Gb
**VCF**	Variant genotypes	57 Mb
**gVCF**	Variant genotypes + invariant region blocks	1.3 Gb
**eVCF**	Variant + dbSNP 141 genotypes	75 Mb

**Table 6:** Compatibility of the eVCF file format with different variation analysis suites.

Software	% genotypes imported	% homozygous reference genotypes imported
**KnoSys 3.1**	99.9%	99.9%
**SVS 8.2.1**	99.9%	99.9%
**ANNOVAR 2014Nov12**	99.9%	99.9%
**VEP (v. 77)**	99.9%	99.9%

**Table 7:** Comparison of the number of dbSNP, ClinVar and GWAScat sites represented using VCF, gVCF and eVCF files.

	VCF	gVCF	eVCF
**dbSNP**	3,344,185	49,704,534	49,704,534
**ClinVar**	1,810	83,675	83,675
**GWAScat**	6,700	13,357	13,357

## Discussion

VCF is a generic file format for storing DNA sequence variants relative to a genome reference sequence, which was developed in the 1000 Genome Project [18,29,30]. The VCF format is compact and scalable, allowing rapid data retrieval for variants in a range of genomic positions, and it has quickly become the *de facto* standard for the storage of variants data. It has therefore been adopted by other projects (UK10K, dbSNP and NHLBI Exome Project) and by most variant-calling pipelines [19,31,32]. Although the VCF format matches the needs of projects aiming to discover polymorphisms in human populations, it was not designed to represent clinically-relevant homozygous reference regions.

We found that more than 88,428 rare variants (MAF < 1% in the general population) are represented in the human reference genome and are consequently not reported in a standard VCF file except when observed heterozygously with another allele. Consistent with the view that functional allelic variants are subject to purifying selection [33–35], rare variants may be particularly relevant during the clinical assessment of common and rare diseases [36,37]. The hg19 genome also carries some well-known pathogenic variants annotated in ClinVar, including rs6025, which causes an Arg506Gln substitution (relative to the common “variant” allele) in the gene *F5* that prevents the inactivation of factor V by protein C thus increasing the risk of thrombosis [38], and rs4784677, a rare variant that causes an Asn70Ser substitution in the gene *BBS2* identified as a putative causative mutation in individuals affected by Bardet-Biedl syndrome, a genetically heterogeneous disorder characterized by pigmentary retinal dystrophy, polydactyly, obesity, developmental delay and renal defects [39].

Many disease-relevant variants reported during the last 10 years of research have been identified by SNP genotyping in GWAS experiments, including late-onset Alzheimer’s disease [40], Crohn’s disease [41], coronary artery disease [42], hyperlipidemia [43] and cancer [44]. The hg19 sequence carries many risk alleles, including the A allele for rs956225, which is strongly associated (OR = 3.3) with Alzheimer’s disease in African-Americans, and the T allele for rs527409, a locus on chromosome 1p31 strongly associated (OR = 2.9) with Kawasaki Disease, an acute self-limited vasculitis of infants and children that manifests as fever and signs of mucocutaneous inflammation. Furthermore, hg19 carries several risk alleles detected by GWAS that are associated with adverse responses to chemotherapy in breast cancer (e.g. rs10818894, rs10443215) and acute lymphoblastic leukemia (rs6971925, rs7128311). The lack of information about homozygous reference genotypes in the VCF file for individual patients is therefore a limitation of the standard variant analysis because it does not investigate clinically-relevant pathogenic or response-related variants represented in the reference genome.

It’s worth noting that some of the genomic sites corresponding to well known variants associated to disease and represented by the pathogenic allele in the hg19 sequence, such has the causal variant of Factor V Leiden trombophilia (rs6025) [38], have been corrected in the hg38 reference sequence to represent the non pathogenic allele. Moreover, the reference allele has been substituted by the major allele in the latest genome release (hg38) in 9.4% of known variant sites represented by a minor allele (MAF <1%) in the hg19 sequence (dbSNP 141). However, the majority (∼91%) of the genomic sites represented by potentially clinically relevant sites in the reference sequence, such as rare (<1%), risk or pathogenic alleles, are unchanged in the hg38 release compared to the hg19 genome. Furthermore, a large part of the scientific community is still using the hg19 genome waiting for a migration of all the tools and databases to the hg38 genome release.

We applied the standard variant calling approach to WGS data in a clinical context for a patient affected by abnormal electrocardiac activity and found that it was unable to provide information about sites genotyped as homozygous references, preventing access to genotype information for 41 of 96 SNPs covered by the commercial MI Risk plus kit, for 93% of the SNPs present in dbSNP build 141, and for 49% of the SNPs represented in the GWAS catalog. Such no-variant calls could be inferred as homozygous reference sites, but they may also represent poor read coverage or low mapping quality [45,46]. Neglecting to investigate the genotype call for known risk alleles might cause relevant variants to be overlooked, preventing the identification of genetic predispositions to disease.

In a previously proposed solution heuristic filters such as the absence of known structural variants and integration of sequencing datasets from different platforms have been used to make high confidence SNP, indel and homozygous reference genotype calls [47]. However, this approach is not widely applicable and does not calculate a per-base confidence score for homozygous reference calls. We therefore adopted the recently-proposed gVCF file format (https://sites.google.com/site/gvcftools/), an extension to the standard VCF format supported by most recent variant calling software such as Illumina’s variant calling pipeline Isaac [19] and the Genome Analysis Tool Kit suite (GATK; versions 2.7 and above) developed by the Broad Institute [31], which store genotypes of variant sites and invariant genomic regions with associated calling scores. The gVCF-based approach proved to be more comprehensive than standard approaches based on VCF format because it allowed all 96 sites interrogated by MI Risk plus to be genotyped as well as the majority of SNPs present in dbSNP and the GWAS catalogs. The genotype calls from the gVCF file also allowed us to identify 96 exonic homozygous reference variants with a allele frequency < 5% and 57 with a frequency < 1%, and doubled the number of SNPs associated with a phenotype by GWAS by adding 6657 homozygous reference sites that were not detected by classical variant analysis. Among the homozygous reference SNPs genotyped in the patient and associated with a phenotype by GWAS, we identified 429 sites homozygous for the risk allele and 1036 sites homozygous for the protective allele, accounting for 307 and 511 phenotypes, respectively. Risk alleles showed odds ratios up to 36 and protective alleles showed values as low as 0.01, indicating strong associations with the phenotype. If we had assumed that a no call corresponds to a homozygous reference call as in standard variant analysis, we would have overestimated by 3,964,772 the number of homozygous reference sites corresponding to known SNPs. Furthermore, because 5280 of these variants (132 located in exons) were represented in the reference genome by rare alleles (MAF < 1%), of which 3 were annotated as pathogenic variants in ClinVar and 17 were annotated as risk alleles detected by GWAS, the standard approach based on the VCF format would have missed some potentially relevant information for the clinical evaluation of the genomic data.

Standard variant analysis showed that the patient is heterozygous for the pathogenic rs199473022 T allele, a rare variant causing a Gly1036Asp substitution in the ion channel encoded by the gene *KCNH2*. This was first identified in one of 541 Long QT Syndrome (LQTS) patients, but not in any of 750 controls [48]. The same variant was also reported in an individual who was resuscitated from cardiac arrest [49]. This variant alters the biophysical properties of the KCNH2 channel, causing statistically significant reductions in ionic current and accelerated fast-phase deactivation compared to normal KCNH2 [49]. The patient was found also to be heterozygous for SNP rs6795970, a non-synonymous variant in the gene *SCN10A* encoding a sodium channel isoform expressed in cardiac neurons, myocardium and the specialized conduction system, indicating a role in cardiac electrophysiological function [50–52]. The rs6795970 SNP is represented by risk allele A in the reference genome and associated with variability in the PR interval and QRS duration [53]. These findings correlate with the clinical phenotype observed in the patient, i.e. abnormal electrical activity characterized by a long QT interval. However, the analysis of invariant sites using the gVCF file showed that the patient is also homozygous reference for the risk allele T at site rs10428132, which is located in the gene *SCN10A* and that, according to GWAS data, is the locus most strongly associated (OR = 2.55; P = 1 x 10^-68^) with Brugada syndrome, a rare disease with a high risk of sudden cardiac death reflecting uncoordinated electrical activity in the lower chambers of the heart [54]. This result, which adds further support to the genetic basis of the phenotype observed in the patient, would not have been achieved using a standard variant calling pipeline.

Given the above result, we foresee (and suggest) the widespread adoption of the gVCF format in clinical genome sequencing. However, the gVCF format is not yet supported by current variant analysis platforms such as Annovar, SVA and PLINK/Seq, it does not match the scalability of the VCF format because it does not support multiple samples, and the files generated from WGS data tend to be large and thus more difficult to store, transfer and search than VCF files. With the rapid adoption of cloud storage and cloud-based bioinformatic analysis, the adoption of larger files means an increase in the time required to transfer and analyze data, and an increase in storage costs. Therefore, we propose the adoption of an extended VCF (eVCF) format containing both the genotypes of variants as a standard VCF, plus the genotypes of a set of sites of interest for the clinical evaluation. This can be processed with standard annotation and analysis platforms designed for the VCF format while retaining the compact size of standard VCF files (Table 5). Like the gVCF format, eVCF files contain the information relevant to all clinical variants of interest allowing a much more comprehensive evaluation of disease risk.

An eVCF file can be generated at the variant calling step by providing a list of genomic sites of interest to a variant caller such as samtools [55]. However, genotype calling must be repeated from the original alignment files to maintain an updated reference list of variants whenever new SNPs have to be interrogated. Regeneration of the eVCF file from the parental gVCF allows to easily extract genotypes from new sites of interest without having to reprocess the storage and computationally demanding alignment files. Nevertheless, by matching the performance of the gVCF format while remaining compatible with VCF-based applications, the eVCF format clearly outperforms VCF and should be adopted as the format of choice at least until the gVCF format is supported by most popular variant analysis platforms.

## Supporting Information

S1 Fig Uniformity of genome coverage. Distribution of genome sequence coverage across the unmasked genome for datasets produced with standard library preparation protocol involving a PCR enrichment (STD) and with a PCR-Free library preparation protocol (PCRFree).
(PDF)

S2 Fig Coverage improvement of difficult regions in PCRFree dataset. Percentage average read depth improvement of high GC regions (100 bp with ≥ 75% GC content), huge GC regions (100 bp ≥ 85% GC content) and AT dinucleotides (≥ 30 bp of repeated AT dinucleotides) with PCR-Free protocol with respect to the standard protocol with PCR enrichment.
(PDF)

S3 Fig Coverage of an high GC region by STD and PCRFree datasets. Integrative Genomics Viewer (IGV) screenshot of STD and PCRFree alignments in a region with high GC content. HighGC and HugeGC tracks show regions with ≥ 75% GC content and ≥ 85% GC content respectively. Gaps in STD and Gaps in PCRFree tracks shows regions longer than 10bp with a low read depth (read depth < 5), low alignment score (Q score < 10) and low basecall quality (Q < 10) in STD and PCRFree datasets respectively.
(PDF)

S4 Fig Comparison of HumanOmni 2.5M microarray (HO2.5) with sequencing. Number of HumanOmni 2.5M sites genotyped by microarray analysis and WGS.
(PDF)

S5 Fig Location of SNPs or INDELs relative to the probed base in HumanOmni2.5 array. Histogram showing number of SNPs or INDELS at different positions relative to the probed SNP on the Illumina HumanOmni2.5 array.
(PDF)

S1 Table Genotyping of 96 SNPs related to myocardial infarction by MI Risk Plus kit and WGS. (XLSX)

S2 Table Genotypes of 2040 sites carrying a protective allele on the hg19 reference genome genotyped by WGS. (XLSX)

S3 Table Genotypes of 1025 sites carrying a risk allele on the hg19 reference genome genotyped by WGS. (XLSX)

S4 Table Genotyping of known variants (dbSNP 141 + Clinvar) with a minor allele on reference (MAF < 50%) by WGS. (ZIP)

S5 Table Genotyping of known variants (dbSNP 141) with a minor allele on reference (MAF < 5%) by WGS. (XLSX)

S6 Table Genotyping of ClinVar pathogenic and drug response variants by WGS. (XLSX)
