# Supplementary figures and images for: The Use of Non-Variant Sites to Improve the Clinical Assessment of Whole-Genome Sequence Data
Source: PLoS One. 2015 Jul 6;10(7):e0132180. doi: 10.1371/journal.pone.0132180 (PMC4492948; doi:10.1371/journal.pone.0132180)

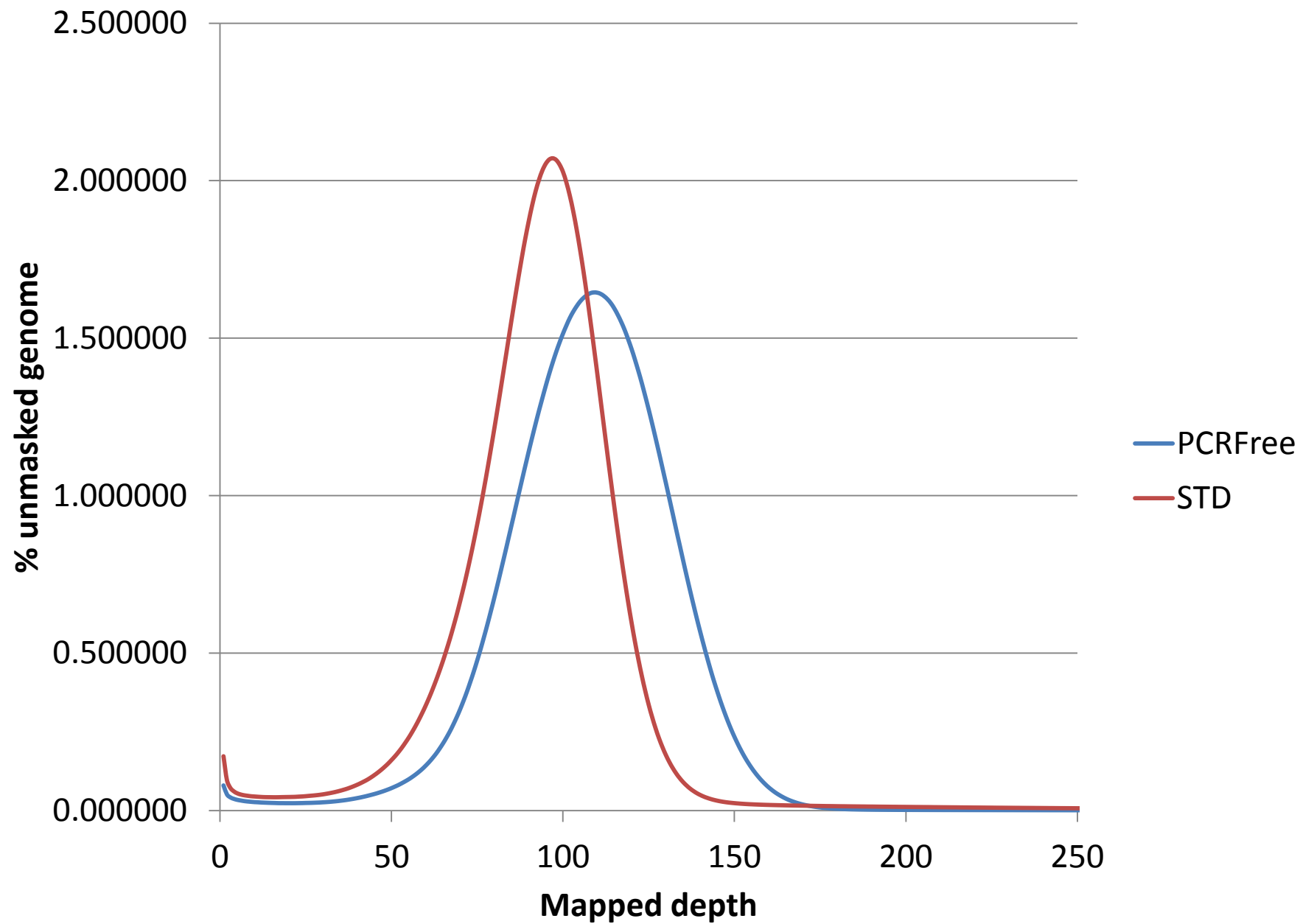

Supplement: S1 Fig — Distribution of genome sequence coverage across the unmasked genome for datasets produced with standard library preparation protocol involving a PCR enrichment (STD) and with a PCR-Free library preparation protocol (PCRFree). (PDF) [file pone.0132180.s001.pdf]

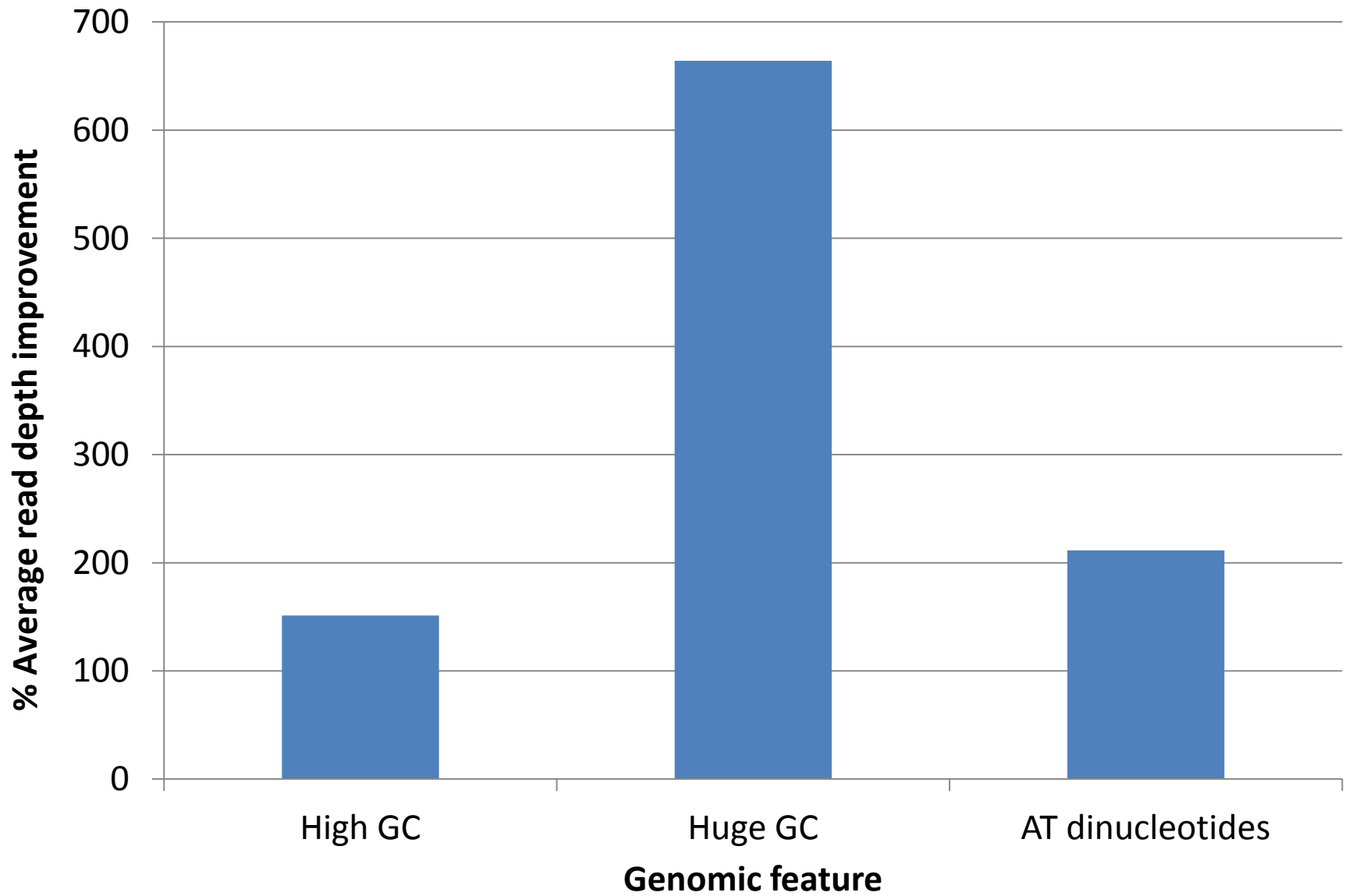

Supplement: S2 Fig — Percentage average read depth improvement of high GC regions (100 bp with ≥ 75% GC content), huge GC regions (100 bp ≥ 85% GC content) and AT dinucleotides (≥ 30 bp of repeated AT dinucleotides) with PCR-Free protocol with respect to the standard protocol with PCR enrichment. (PDF) [file pone.0132180.s002.pdf]

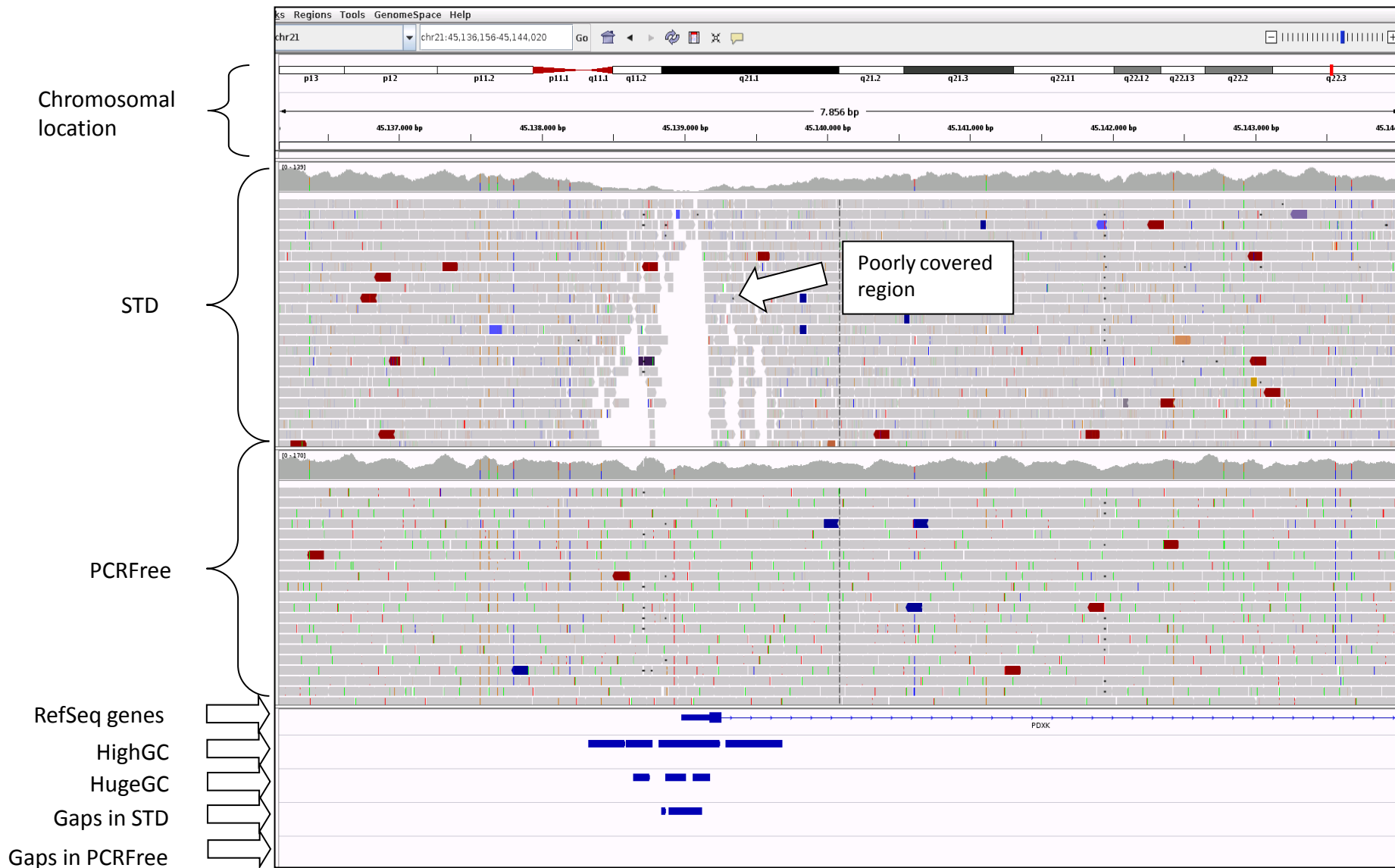

Supplement: S3 Fig — Integrative Genomics Viewer (IGV) screenshot of STD and PCRFree alignments in a region with high GC content. HighGC and HugeGC tracks show regions with ≥ 75% GC content and ≥ 85% GC content respectively. Gaps in STD and Gaps in PCRFree tracks shows regions longer than 10bp with a low read depth (read depth < 5), low alignment score (Q score < 10) and low basecall quality (Q < 10) in STD and PCRFree datasets respectively. (PDF) [file pone.0132180.s003.pdf]

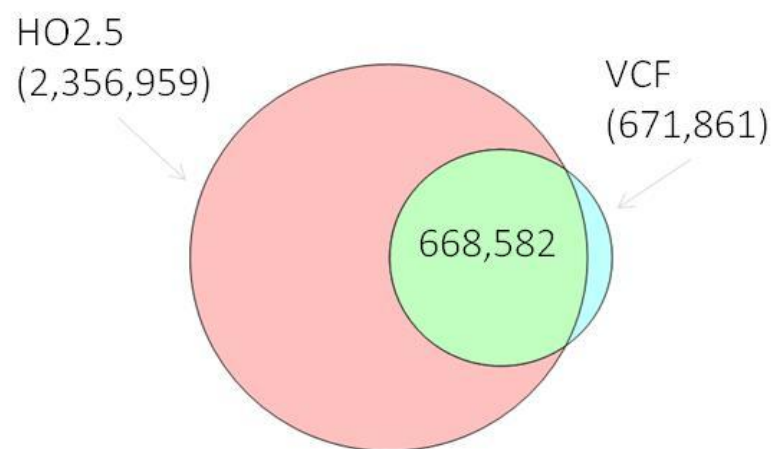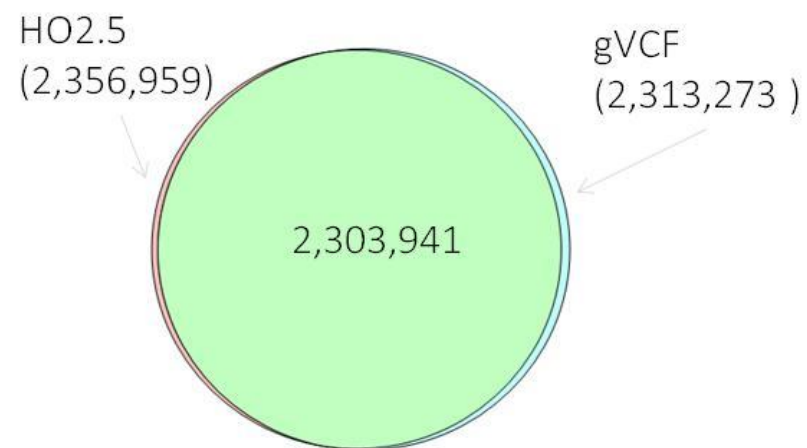

Supplement: S4 Fig — Number of HumanOmni 2.5M sites genotyped by microarray analysis and WGS. (PDF) [file pone.0132180.s004.pdf]

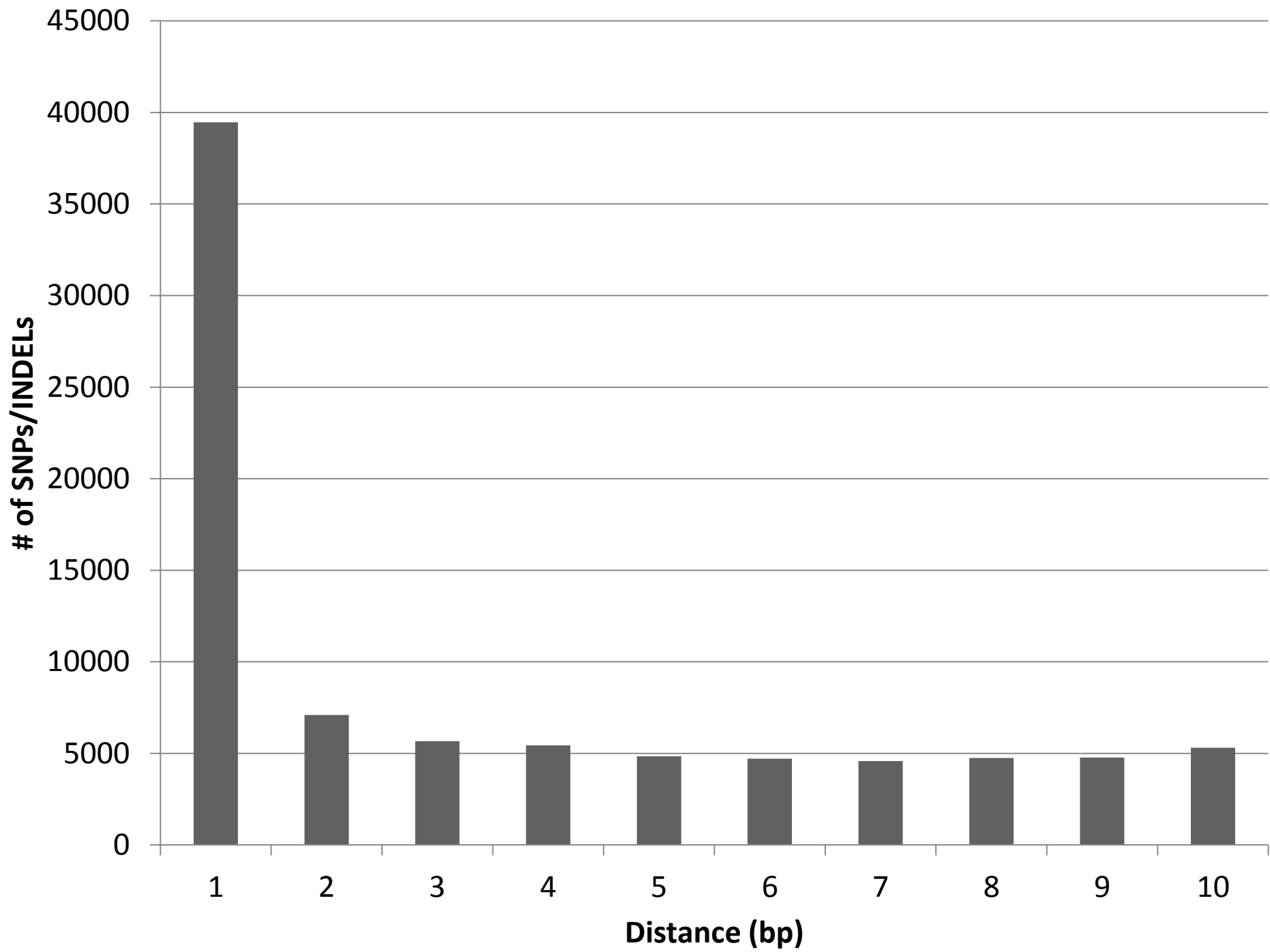

Supplement: S5 Fig — Histogram showing number of SNPs or INDELS at different positions relative to the probed SNP on the Illumina HumanOmni2.5 array. (PDF) [file pone.0132180.s005.pdf]
